# Editorial for the Special Issue on Micro and Smart Devices and Systems

**DOI:** 10.3390/mi14010164

**Published:** 2023-01-08

**Authors:** Zebing Mao, Jin Xie, Hong Ding

**Affiliations:** 1 Department of Engineering Science and Mechanics, Shibaura Institute of Technology, 3-7-5, 02-C-25b Toyosu, Koto-ku, Tokyo 135-8548, Japan; 2 The State Key Laboratory of Fluid Power and Mechatronic Systems, Zhejiang University, Hangzhou 310027, China; 3 Department of Biomedical Engineering, The University of Texas at Austin, Austin, TX 78712, USA

Micro and smart devices and systems are small, interconnected devices and systems that are designed to be highly functional, efficient, and convenient. These devices and systems often use advanced technologies such as microelectromechanical systems (MEMS), the Internet of Things (IoT), and artificial intelligence (AI) to perform tasks and functions that can be controlled and accessed remotely [1,2,3]. Examples of micro and smart devices and systems include Microcontrollers, Internet of Things devices, and Wearable devices. Microcontrollers [4] are small, low-power computer systems-on-a-chip that can be used to control devices and systems. They are often used in applications such as home appliances, automotive systems, and industrial control systems. Internet of Things (IoT) devices [5] are devices that are connected to the internet and can collect and transmit data. They can include a wide range of devices, such as smart thermostats, smart appliances, and smart sensors. Wearable devices [6] are devices that can be worn on the body, such as smartwatches, fitness trackers, and smart glasses. They often have sensors and other technologies that allow them to collect data about the wearer’s activity and environment. They are an increasingly important part of the modern world and are expected to continue to grow and evolve in the coming years. In our special issue, we will also focus on the devices which are still under investigation. Some examples include solid/fluidic actuators and sensors, functional fluids, microfluidics, self-actuation/sensing, self-power systems, self-oscillating, smart hydrogels, intelligent control systems, lenses, origami batteries, fuel cells, etc. We believe they will have great protentional in various fields of robotics, telecommunications, chemistry, and biology. We invited renowned scientists in the field to share their expertise and perspectives, and collected 15 papers in our special issue, in the following fields: electrophoretic displays, solar cells, transducers, fluidic systems, electronic components, etc.

As one type of smart system, three-color electrophoretic displays are a type of display technology that uses electrophoresis, a process in which charged particles are moved through a liquid or gel under the influence of an electric field, to create an image. One advantage of electrophoretic displays is that they have very low power consumption since they only require electricity to change the image and do not require a continuous power supply to maintain it. However, the response time of three-color EPDs increases with the addition of red particles. Hu Zhang et al. [7] proposes a new driving waveform based on high-frequency voltage optimization and electrophoresis theory to shorten the response time. The proposed driving waveform consists of an activation stage, a new red driving stage, and a black or white driving stage. By eliminating an erase stage, the response time of the particles is effectively reduced. The results show that the proposed driving waveform has a better performance compared to the traditional driving waveform. Another example is solar cells. Traditionally, dye-sensitized solar cells (DSSCs) use expensive and rare element-containing FTO/ITO as the electrode, which is difficult to meet the requirements of flexibility. Jianjun Yang et al. [8] proposes a new flexible DSSC structure with a rare-element-free all-metal electrode. First, a photoelectrode with small holes is prepared on the black light cathode to achieve a continuous oxidation-reduction reaction in the electrolyte. Second, the processing technology of porous titanium dioxide (TiO_2_) films are analyzed. By testing the J-V characteristics, it is found that the performance is better when the heating rate is slower. Finally, the effects of different electrode material combinations are compared by experiment.

We also collected some recent research about ultrasonic transducers. Ultrasonic transducers are devices that convert electrical energy into mechanical vibrations or sound waves, or vice versa. Ultrasonic transducers can be classified into two main types: piezoelectric and magnetostrictive. Piezoelectric microelectromechanical ultrasonic transducers (PMUTs) are a promising alternative to traditional large-block piezoelectric ceramics-based ultrasonic transducers. However, the reported emission sensitivities of PMUTs are far from satisfactory. Mengjiao Qu et al. [9] report a beam-membrane-coupled PMUT (BM-PMUT) that improves the emission sensitivity by simultaneously increasing the sound emission area and maintaining comparable vibration amplitudes. In their report, BM-PMUTs achieve a piston-like mode shape, leading to approximately a twofold improvement in effective sound emission area compared to traditional T-PMUTs of similar size. Due to the larger sound emission area and comparable vibration amplitudes, the normalized far-field sound pressure level of BM-PMUTs is 8.5 dB higher than that of T-PMUTs. Also, Omer M. O. Abdalla et al. [10] present a numerical reduced-order modeling (ROM) method for complex multi-layered piezoelectric microelectromechanical ultrasonic sensors (PMUTs) arrays. Numerical modeling techniques are used to generate PMUT arrays consisting of a considerable number of sensors, which can greatly reduce the computational cost without compromising accuracy. The modeling approach is based on the coupling of shell elements applied to the PMUT structural layer and three-dimensional solid elements applied to the piezoelectric layer.

Fluidic systems including microfluidics, droplet networks, and hydraulic components are attractive in recent years since they involve the flow of fluids, such as gases or liquids. These systems can be found in a wide range of applications, including transportation, energy production, and manufacturing [11,12]. First, Nassim Rousset et al. [13] conducts a comprehensive review of the microfluidic droplet networks. The droplet networks have multi-functional configurations and air-liquid interfaces (ALIs). These ALIs provide ample oxygen, fast liquid turnover, passive degassing, and liquid phase stability through capillary pressure. Liquid-electrical analogies are effective "first-hand" methods for exploring the design and operational parameter space of microfluidic droplet networks. However, due to the nonlinear nature of capillary pressure at the ALI, there is no direct electrical analogy for droplets. Nassim Rousset et al. [13] present a circuit-based model for suspended and standing droplet junctions. They show a phase diagram describing the nonlinear capillary pressure of suspended droplets and propose a method to find the flow rate and pressure within droplet networks. On the other hand, Jia Man et al. [14] use microfluidic technology to treat Acute kidney injury (AKI) via intravenous injection. Previous studies have shown that histone deacetylase inhibitors (HDACi) can attenuate renal damage and promote renal recovery in AKI. However, the hydrophobic nature of HDACi, such as vorinostat (SAHA), requires organic solvents to facilitate dissolution, resulting in unavoidable adverse effects. Jia Man et al. [14] prepare calcium alginate microspheres (CAM) as carriers of HDACi via microfluidics for the treatment of AKI via intravenous injection. In addition, they select CAM with a suitable size as the carrier of HDACi and deliver the HDACi-loaded CAM into AKI mice via intravenous tail injection. In vivo study results show that HDACi-loaded CAM effectively reduce renal inflammation and relieve. Acharya Phuakrod et al. [15] apply their previously developed semi-automated microfluidic device in combination with our recently developed mini polymerase chain reaction (miniPCR) with a duplex lateral flow dipstick (DLFD) (miniPCR-DLFD) for rapid mass screening and visual species identification of lymphatic filariae in human blood. In the study, the researchers use 20 human blood samples tested positive for Malayi microfilariae, 14 human blood samples tested positive for Bancroftian microfilariae, and 100 human blood samples tested negative for mf. The researchers use microfluidic devices to detect and visually identify the species of mf. To identify the species of mf trapped in the microfluidic chips, the researchers extract the DNA of the mf and amplify it using miniPCR, and then analyze it using DLFD. The researchers also use thick blood smear staining as a gold standard technique to detect microfilariae. The results of the study show that the microfluidic devices and miniPCR-DLFD platform are able to consistently and accurately detect and identify microfilariae, compared to the gold standard thick blood smear technique. The microfluidic devices, miniPCR, and DLFD are portable and do not require additional equipment. The researchers believe that using this screening and visual identification platform can provide reliable, economical, and rapid monitoring for the presence of Lymphatic filariasis (LF) infections in resource-limited environments. Ane Larrea et al. [16] report a simple and robust microfluidic method for producing Trojan particle systems (microparticle systems) of nanoparticles as oral active drug ingredient carriers. The microfluidic system is based on two co-axial capillaries and produces single dispersed water-in-oil-in-water (W/O/W) double emulsion droplets in a highly controlled manner and accurately controls the particle structure produced, including the core and shell size. The effect of the three-phase flow rate, pH, and drying process on the formation and overall size is evaluated. The droplets are then used as templates to produce pH-sensitive Trojan particles upon solvent evaporation. The Trojan particles’ shells are made of Eudragit^®^, a copolymer of methacrylic acid-ethyl acrylate, which will allow the Trojan particles’ payload to pass through the stomach undegraded and then dissolve in the intestinal fluid to release the internal payload. They further demonstrate that Trojan particles release embedded PLGA nanoparticles upon contact with the appropriate medium, demonstrating their potential as oral drug delivery systems. Furthermore, Meisheng Yang et al. [17] introduce a smart valve to our special issue. In the past, researchers have largely simulated and carried out sporadic experimental studies on the static and dynamic characteristics of digital valves, but have not systematically and deeply studied the characteristics of digital valves. Based on the principles and functions of valves and the experimental system, the author conducts experimental studies on three static characteristics, including pressure drop-flow rate characteristics, signal-pressure characteristics, and signal-flow rate characteristics, under various variables. Through this study, relevant performance parameters for using digital valves as system control components in the next step can be obtained, laying the foundation for the accurate control of the system.

Other electronic components are also incorporated in our special issue, e.g., MEMS vapor cells, which are key components for sensors such as chip-scale atomic clocks (CSACs) and magnetometers (CSAMs). Ping Guo et al. [18] propose a new method based on chemical reactions and evaporation for manufacturing wafer-level fillers for MEMS vapor cells. First, Cs metal is obtained by a chemical reaction between cesium chloride and barium nitride in the reservoir bottom plate. Then, the Cs metal is evaporated onto a preform through a microchannel plate and condenses on the inner surface of the glass preform. Finally, the MEMS vapor cell is filled with a buffer gas and sealed by cathode bonding. The results show that the proposed wafer-level filling method for MEMS vapor cells meets the requirements of CSAC and other sensors. Another example is the antenna. Sujan Shrestha et al. [19] propose a wideband antenna using three-dimensional printing technology. The antenna is designed using the PREPERM 10 material with a permittivity of 10. The prototype has an overall height of 12.83 mm (0.51 λ) and a lateral dimension of 60 mm × 60 mm and operates at a frequency of 12 GHz (λ = 25 mm). It achieves a wide frequency bandwidth with a voltage standing-wave ratio (VSWR) of less than two, covering the Ku-band from 10 GHz to 15 GHz. The total weight of the system, including the PREPERM 10 and ABS structures with copper-painted prototypes, is 96 g and 79 g, respectively.

Besides the conventional methods, some researchers try to use the recently developed machine learning methods to assist the micro and smart devices and systems. A robotic system that can recognize objects and grasp them autonomously in a cluttered and occluded environment would be highly desirable. Zhongjie Zhang et al. [20] present an autonomous, real-time 6D robotic grasping system that integrates object detection, pose estimation, and grasping planning on a 7-degree-of-freedom (DOF) robotic arm equipped with a low-performance native camera sensor. To estimate the 6D pose of the object, they use the pixel-wise voting network (PV-net). However, the PV-net method is not able to distinguish real objects from their photographs using only RGB images as input. To address this limitation and meet the demands of a real industrial environment, they have developed a rapid analytical method based on point clouds that can determine whether a detected object is real or not. Their system demonstrates stable and robust performance in various installation positions and cluttered scenes. On the other hand, Norah N. Alajlan et al. [21] focus on the Internet of Things (IoT) devices and use machine learning (ML) models to make intelligent decisions. However, these devices often have limited resources, which makes it difficult to execute complex ML models like deep learning (DL) on them. In addition, sending raw data to the cloud for processing can cause delays in system responses, expose private data, and increase communication costs. To address these challenges, a new technology called Tiny Machine Learning (TinyML) has emerged, allowing data to be processed locally on the device without the need to send it to the cloud. TinyML also enables the inference of ML models, including DL models, on devices with limited resources, such as microcontrollers. The goal of this paper is to provide an overview of the development of TinyML and review existing TinyML research. We also analyze the types of ML models used in TinyML studies, the datasets used, and the characteristics of the devices. This review aims to provide a clear understanding of the state of the art and identify future development needs.

Other researchers improved the performance of micro and smart devices and systems via the aspects of materials and chemicals. Samee Azad et al. [22] study the infrared transmission characteristics of VO_2_ thin films synthesized on different substrates using a low-pressure direct oxidation technique. The material quality of these films is found to be high, resulting in significant changes in their electrical and optical properties at the phase transition. When they are illuminated with an infrared (IR) laser at 1550 nm, these films show a change in optical transmissivity of more than 80%. The phase transition in VO_2_ films on transparent quartz and muscovite substrates are triggered by temperature changes induced by a pulsed high-power laser beam and result in modulated IR laser transmission with a low time constant. The effect of mechanical strain on the phase transition in VO_2_ films grown on flexible muscovite substrates is also investigated. The results show that tensile strain shifts the transition temperature to higher values, while compressive strain shifts it to lower values. Additionally, Muhammad Bilal et al. [23] investigate the characteristics of transient, electroviscous, and ternary mixed nanofluids flowing through parallel infinite plates. Ternary mixed nanofluids are synthesized by dissolving titanium dioxide (TiO_2_), aluminum oxide (Al_2_O_3_), and silicon dioxide (SiO_2_) nanoparticles in a carrier liquid of ethanol/water. The goal of the study is to maximize energy and mass transfer rates for industrial and engineering applications. They examine the effects of fluid flow, as well as the additional factors of a magnetic field, exothermic/endothermic reactions, chemical reactions, and activation energy. The flow of ternary mixed nanofluids is modeled using a system of partial differential equations, which is then simplified into a set of ordinary differential equations through similarity substitution. The resulting nonlinear system of dimensionless ordinary differential equations is solved using the parameter continuation method. To validate the results, they compare them with existing studies and provide physical explanations through graphs and tables. The findings indicate that the mass transfer rate increases with increasing Lewis number, activation energy, and chemical reaction values. The velocity and energy transfer rate also increases with the addition of ternary nanoparticles to the base liquid.

In conclusion, micro and smart devices and systems are a rapidly growing and evolving field, with applications in a wide range of industries including healthcare, transportation, manufacturing, and consumer electronics. These systems are characterized by their small size, high level of integration, and ability to perform complex tasks. Some key benefits of micro and smart devices and systems include their ability to save space, reduce energy consumption, improve performance, and increase reliability. They can also be used to gather and process large amounts of data, enabling the development of new applications and services. There are also several challenges associated with the development and deployment of micro and smart devices and systems, including cost, security, reliability, and environmental impact. Despite these challenges, the potential benefits of these systems make them an exciting and important area of research and development.
